# When Gas Replaces the Liver: A Rare Case of Successfully Treated Emphysematous Hepatitis

**DOI:** 10.1155/crgm/5558002

**Published:** 2025-11-11

**Authors:** Dirin Ukwade, Hasan S. Raza, Omar T. Ahmed, James S. Love, Maria El Gemayel, Jamie L. Berkes

**Affiliations:** ^1^Department of Medicine, University of Illinois, Chicago, Illinois, USA; ^2^Division of Gastroenterology and Hepatology, Department of Medicine, University of Illinois, Chicago, Illinois, USA

**Keywords:** emphysematous hepatitis, gas-forming liver infection, percutaneous drainage

## Abstract

Emphysematous hepatitis is a rare condition characterized by the presence of gas within the hepatic parenchyma. Only a limited number of cases have been reported, with most patients experiencing poor outcomes and median survival time ranging from hours to days after diagnosis. We present a case of a patient with uncontrolled diabetes, abdominal pain, and fatigue who was found to have emphysematous hepatitis. The patient was successfully treated with antibiotics and percutaneous drainage without the need for surgical intervention. This case represents the second reported instance of survival following percutaneous drainage, highlighting the potential efficacy of this minimally invasive approach.

## 1. Introduction

Emphysematous hepatitis (EH) was first described by Blachar et al. in 2002 as a condition characterized by hepatic parenchymal destruction with the presence of gas on imaging [1]. The clinical presentation of EH is variable, with common symptoms including fever and abdominal pain. In severe cases, patients may present with confusion and hypotension. Laboratory findings often reveal elevated liver enzymes and leukocytosis, indicative of an underlying infection. Due to its rarity and nonspecific presentation, EH requires a high index of suspicion for timely diagnosis. Early identification is critical, as the disease is associated with high mortality, with most reported cases resulting in fatality within hours to days of diagnosis [1–14]. Here, we present the second known case of EH successfully treated with antibiotics and percutaneous drainage.

## 2. Case Report

A 59-year-old male with a medical history significant for uncontrolled Type 2 diabetes mellitus, hyperlipidemia, and hypertension presented to an outside hospital with a 4-day history of abdominal pain and fatigue. Initial laboratory studies revealed leukocytosis (12.3 × 10^3^/L), elevated liver enzymes (aspartate aminotransferase [AST] 399 U/L, alanine aminotransferase [ALT] 339 U/L, and alkaline phosphatase [ALP] 268 U/L), total bilirubin of 1.4 mg/dL, lipase of 54 U/L, and an international normalized ratio (INR) of 1.2. During his hospital course, his white blood cell count peaked at 18.9 × 10^3^/L, and his AST and ALT levels rose to 1650 and 1321 U/L, respectively.

**Figure 1:**
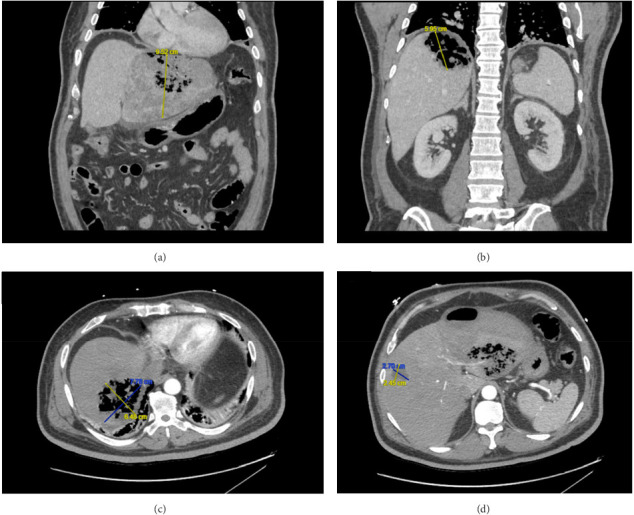
CT of the abdomen with IV contrast. (a) Coronal view of gas occupying the left hepatic lobe measuring 14.4 × 10.2 × 9.5 cm. (b) Coronal view of an additional similar collection within the right hepatic lobe measuring 7.8 × 6.5 × 5.95 cm. (c) Axial view of an additional similar collection within the right hepatic lobe measuring 7.8 × 6.5 × 5.95 cm. (d) Axial view of rim-enhancing collection in the right hepatic lobe measuring 2.8 × 2.7 × 2.45 cm.

The patient was transferred to our institution for evaluation for liver transplantation. Further hepatology workup included viral hepatitis serologies, acetaminophen level, and antinuclear antibodies (ANA), which were all negative. Broad-spectrum antibiotics were initiated empirically. Blood cultures subsequently grew *Klebsiella pneumoniae*. A contrast-enhanced computed tomography (CT) scan of the abdomen revealed extensive gas-forming collections in both the left and right hepatic lobes, consistent with EH. A focal hypodensity in the mid-right hepatic lobe was also noted, likely representing an additional site of infection (Figure 1).

Given the previously identified collections on CT, the decision was made to pursue a CT-guided percutaneous aspiration and drainage of the hepatic collections to also assess for possible concomitant pyogenic liver abscess (PLA). Cultures of drained fluid also grew *K. pneumoniae*. The drains remained in place for approximately 2 months, with interval imaging demonstrating gradual resolution of the collections.

## 3. Discussion

Since the initial description by Blachar et al. in 2002, EH has remained an exceedingly rare condition, with only a handful of cases reported in the literature [1, 6, 15]. The majority of patients with EH have a history of uncontrolled diabetes mellitus, as seen in our case.

The pathophysiology of EH remains poorly understood. Current evidence suggests that EH represents a necrotizing infection of the liver parenchyma, with the most commonly implicated organisms being *K. pneumoniae*, *Clostridium perfringens*, *Escherichia coli*, and *Enterococcus faecalis*. The pathogenesis has been proposed to possibly be similar to that of other emphysematous infections, where the high glucose tissue concentration that is seen in diabetic patients attracts organisms that then ferment the sugar into carbon dioxide and leads to the production of gas within the hepatic tissue [13]. However, this hypothesis does not fully explain cases occurring in nondiabetic individuals, suggesting that additional factors, such as impaired hepatic perfusion or immunodeficiency, may play a role.

The diagnosis of EH relies on imaging, particularly contrast-enhanced CT, which demonstrates gas within the liver parenchyma. The condition is often associated with compromised arterial and portal venous blood supply, resulting in infarcted liver segments that appear as nonenhancing, hypodense areas on imaging [16]. It is also important to differentiate EH from gas-forming PLA, which also presents with hepatic gas. However, PLA is characterized by pus-filled fluid collections rather than parenchymal destruction [1].

Timely diagnosis and intervention are critical in EH, as the condition is associated with high mortality. Most reported cases have resulted in death within hours to days despite prompt antibiotic therapy (Table 1) [1–14]. To date, only seven cases of survival have been documented, including the present case [15–20]. In all surviving cases, antibiotic therapy was combined with either surgical or percutaneous drainage (Table 1). Notably, only two cases (including ours) have reported survival following antibiotic therapy and percutaneous drainage alone [18]. While some authors advocate for surgical drainage as the definitive treatment for EH [16, 19], others have noted that the available data are too sparse to establish the superiority of surgical over percutaneous drainage [17]. Our case underscores the potential efficacy of percutaneous drainage as a minimally invasive treatment option and highlights the importance of early intervention in this rare and life-threatening condition. In addition to identifying a rare case of EH that was successfully treated, our case also shows that further research is needed to establish standardized treatment guidelines for EH.

## Figures and Tables

**Table 1:** Summary table describing demographics (age, sex), presence of underlying diabetes, presenting complaint, organism identified, and treatment of previously reported cases of emphysematous hepatitis with emphasis on outcomes of each treatment of previously reported cases (excluding ours).

Reference (year)	Age	Sex	Diabetes present?	Presenting complaint	Organism	Treatment	Outcome
*Diabetic with Klebsiella*							
Blachar et al. (2002) [1]	43	F	Yes	Abdominal pain, fever, multiorgan failure	*Klebsiella pneumoniae*	Antibiotics + Percutaneous drainage	Fatal
Porez et al. (2023) [17]	59	M	Yes	Fever, confusion	*Klebsiella pneumoniae*	Antibiotics + Surgery (exploratory laparotomy and drain placement)	Survival
Pan et al. (2023) [18]	48	M	Yes	Nausea, vomiting, diarrhea, fever, abdominal pain	*Klebsiella oxytoca*	Antibiotics + Percutaneous drainage	Survival

*Nondiabetic or unknown diabetes status with Klebsiella*							
Bofill et al. (2021) [2]	82	M	No	Fever, confusion, RUQ pain	*Klebsiella pneumoniae*	Antibiotics + Percutaneous drainage	Fatal
Lin et al. (2012) [3]	59	F	Glucose intolerant but no formal diagnosis of DM	Nausea, abdominal pain, hypotension	*Klebsiella pneumoniae*	Antibiotics only	Fatal

*Diabetic with Clostridium perfringens*							
Bayerl et al. (2023) [19]	79	M	Yes	Fever, shortness of breath	*Clostridium perfringens*	Antibiotics + Surgery (exploratory laparotomy and drain placement)	Survival
Calderon and Serfin (2020) [4]	80	F	Yes	Abdominal pain, nausea	*Clostridium perfringens*	Unclear, due to quick deterioration (no intervention referenced)	Fatal

*Nondiabetic or unknown diabetes status with Clostridium septicum*							
Al Khatib et al. (2024) [5]	70	F	No	Abdominal pain, hematochezia, hypotension	*Clostridium septicum*	Antibiotics + Percutaneous drainage + Surgical resection of additional cecal tumor	Fatal

*Diabetic with Escherichia coli*							
Ayoub et al. (2024) [6]	77	F	Yes	Fever, abdominal pain	*Escherichia coli*	Antibiotics only	Fatal

*Nondiabetic or unknown diabetes status with Escherichia coli*							
Miranda et al. (2020) [7]	74	M	No	Fever, AMS, productive cough, abdominal pain	*Escherichia coli*	Antibiotics only	Fatal
Estébanez-Ferrero et al. (2021) [20]	67	F	No	Abdominal pain, vomiting	*Escherichia coli*	Antibiotics + Surgery (exploratory laparotomy and drain placement)	Survival
Tekinhatun and Yavaş (2024) [8]	80	F	No	Fever, AMS, tremors, aphasia, abdominal pain, nausea, vomiting, hypotension	*Escherichia coli*	Antibiotics only	Fatal

*Diabetic with multiple infections*							
Francois et al. (2022) [15]	70	F	Yes	Epigastric pain	*Escherichia coli, Streptococcus anginosus, and Klebsiella oxytoca*	Antibiotics + Percutaneous drainage + Surgical drainage	Survival
Ghosn et al. (2019) [16]	38	F	Yes	Fever, nausea, vomiting, abdominal pain with guarding	*Escherichia coli and Enterococcus faecium*	Antibiotics + Surgical drainage	Survival

*Nondiabetic or unknown diabetes status with multiple infections*							
Kim et al. (2012) [9]	80	F	No	RUQ pain	*Clostridium perfringens and Escherichia coli*	Antibiotics + Percutaneous drainage	Fatal
Létourneau-Guillon et al. (2010) [10]	53	M	No	Fever, chills	*Enterobacter cloacae and Clostridium perfringens*	Antibiotics only	Fatal
Azri et al. (2020) [11]	75	F	No	Fever, abdominal pain	*Klebsiella pneumoniae, Escherichia coli, Enterococcus faecalis, Clostridium perfringens*, *and Aeromonas ichthiosmia*	None reported	Fatal
Nada et al. (2017) [12]	73	F	No	AMS, hypoglycemia	*Streptococcus mutans and Enterococcus faecalis*	Antibiotics only	Fatal

*Diabetic with no identified infectious source*							
Chauhan et al. (2022) [13]	77	M	Yes	AMS, hypotension	None identified	Antibiotics + Percutaneous drainage	Fatal
Dimitriou et al. (2014) [14]	72	M	Yes	RUQ pain, fever, chills	None identified	Antibiotics only	Fatal

Abbreviations: AMS, altered mental status; DM, diabetes mellitus; RUQ, right upper quadrant.
